# Meta-Analysis of Pulmonary Transcriptomes from Differently Primed Mice Identifies Molecular Signatures to Differentiate Immune Responses following* Bordetella pertussis* Challenge

**DOI:** 10.1155/2017/8512847

**Published:** 2017-01-24

**Authors:** René H. M. Raeven, Jeroen L. A. Pennings, Elly van Riet, Gideon F. A. Kersten, Bernard Metz

**Affiliations:** ^1^Institute for Translational Vaccinology (Intravacc), Bilthoven, Netherlands; ^2^Division of Drug Delivery Technology, Leiden Academic Centre for Drug Research, Leiden, Netherlands; ^3^Centre for Health Protection, National Institute for Public Health and the Environment, Bilthoven, Netherlands

## Abstract

Respiratory infection with* Bordetella pertussis *leads to severe effects in the lungs. The resulting immunity and also immunization with pertussis vaccines protect against disease, but the induced type of immunity and longevity of the response are distinct. In this study the effects of priming, by either vaccination or infection, on a subsequent pathogen encounter were studied. To that end, three postchallenge transcriptome datasets of previously primed mice were combined and compared to the responses in unprimed control mice. In total, 205 genes showed different transcription activity. A coexpression network analysis assembled these genes into 27 clusters, combined into six groups with overlapping biological function. Local pulmonary immunity was only present in mice with infection-induced immunity. Complement-mediated responses were more prominent in mice immunized with an outer membrane vesicle pertussis vaccine than in mice that received a whole-cell pertussis vaccine. Additionally, 46 genes encoding for secreted proteins may serve as markers in blood for the degree of protection (*Cxcl9, Gp2, *and* Pla2g2d*), intensity of infection (*Retnla, Saa3, Il6, *and* Il1b*), or adaptive recall responses (*Ighg, C1qb*). The molecular signatures elucidated in this study contribute to better understanding of functional interactions in challenge-induced responses in relation to pertussis immunity.

## 1. Introduction

Whooping cough, caused by* Bordetella pertussis*, is an endemic disease with considerable health impact [1, 2]. Several vaccines against* B. pertussis* are marketed or under development. These include whole-cell pertussis vaccine (wPV), acellular pertussis vaccine (aPV), live-attenuated pertussis vaccine (BPZE1), and vaccines based on outer membrane vesicles (omvPV) [3–6]. These vaccines are very different with regard to concept (whole-cell inactivated, subunit, live attenuated), composition, and route of immunization. Although all vaccines confer protection in the mouse challenge model [3–6], the type and level of immunity are different [3–6]. This difference in immunity leads to distinct pulmonary immune responses upon subsequent* B. pertussis* encounter [7, 8].

Analysis of different transcriptome datasets is a tool for unbiased investigation of biological processes. It has been used to compare, for instance, the immune responses induced by different vaccines to identify universal vaccine-induced signatures [9–11]. Previously, differences in pulmonary gene expression of mice immunized with omvPV or wPV [8] and mice receiving a primary infection [7] were elucidated. However, the pulmonary transcriptome datasets obtained by challenge experiments may contain potential gene markers related to pertussis immunity. The identification of those markers may contribute to better understanding of pertussis immunity and as readout in a human challenge model [12].

We performed a meta-analysis of pulmonary transcriptome datasets obtained after a* B. pertussis* challenge in mice with a different pertussis immune status. These included mice with infection-induced immunity, wPV-immunized mice (wPV-mice), omvPV-immunized mice (omvPV-mice), and nonimmunized mice (NI mice) as control [7, 8]. The molecular signatures were characterized with special attention for secreted proteins, since these markers could potentially be useful for monitoring immune responses in blood samples to determine degree of protection, intensity of infection, or promptness of adaptive recall responses for later application in human challenge studies.

## 2. Methods

### 2.1. Datasets

**Figure 1:**
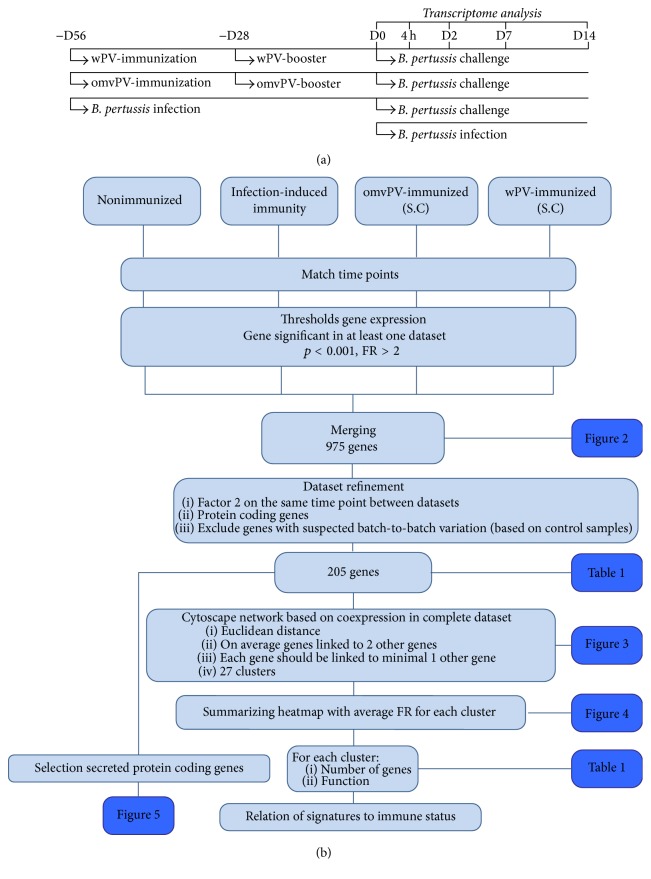
Design and method used in this study. Four transcriptome datasets were included obtained after a* B. pertussis* challenge in the lungs of nonimmunized mice, mice with infection-induced immunity, and mice immunized subcutaneously (SC) with omvPV or wPV. (a) The immunization and challenge scheme of the experiments of these datasets. (b) The different steps of the meta-analysis and criteria used are described and linked to the figures and tables.

We used gene expression datasets from four* B. pertussis* challenge experiments in BALB/c mice. These included data from challenge studies performed 56 days after the primary immunization or infection [7, 8]. We included mice with (i) infection-induced immunity following a single infection with 2 × 10^5^ colony forming units of a* B. pertussis* B1917 strain, mice immunized twice with 4 *μ*g total protein of (ii) wPV or (iii) omvPV, and (iv) nonimmunized mice (Figure 1(a)). The lung colonization data was previously obtained and described for unprimed mice and mice with infection-induced immunity [13] and mice immunized with wPV and omvPV [8].

### 2.2. Gene Expression Analysis

The flow diagram showing all stages of the gene expression analysis and the selection criteria is shown in Figure 1(b). For all datasets, we included five time points: 56 days postprimary infection (p.i.) or immunization, but before challenge (D0), and 4 hours, 2 days, 7 days, and 14 days postintranasal* B. pertussis* challenge (p.c.). Gene expression profiles of nonchallenged and nonimmunized mice were used as a control. In each of the four experiments, differentially expressed genes (DEGs) were identified by using previously described methods [13, 14], namely, a one-way ANOVA at a stringency value of *p* < 0.001 and an absolute Fold Ratio (FR, i.e., challenge response to the control group) ≥ 2.0. Data for the combined set of DEGs (across time points in one study) were merged. This set of DEGs was further refined by (i) identifying DEGs that differed by a fold change ≥ 2.0 across studies at one time point; (ii) excluding genes that are not protein-coding (mainly genes annotated by NCBI as “gene model” or “hypothetical gene”); and (iii) excluding genes with batch-to-batch variation between arrays in the control groups.

### 2.3. Functional Analysis

For the resulting datasets, a coexpression network was created, based on the Euclidean distance between their overall response patterns across all groups and time points. Genes were connected in a network if their coexpression similarity fell in the top 1% of overall most similar responses. Additionally, remaining genes were connected to genes with the most similar response over time in order to include each gene in the network. Further functional analysis and identification of genes that encode for secreted proteins based on the Uniprot-term “secreted” were performed by using DAVID [15].

### 2.4. Data Visualization

Data were visualized using Adobe Illustrator CC 2015, Cytoscape (version 2.8.3) (http://www.cytoscape.org), R (https://www.r-project.org), Genemaths XT (Applied Maths, St-Martens-Latem, Belgium), and Venny (http://bioinfogp.cnb.csic.es/tools/venny/index.html).

## 3. Results and Discussion

### 3.1. Identification of Gene Expression Signature Clusters

**Figure 2:**
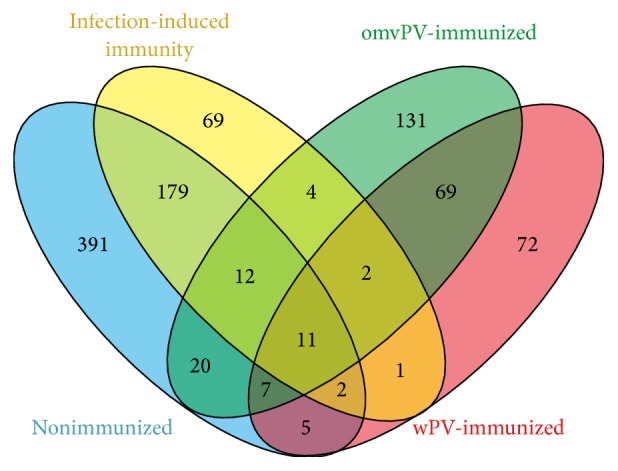
Venn diagram. The overlap of differentially expressed genes between four datasets obtained from lungs of mice at different time points after* B. pertussis* challenge is depicted.

**Figure 3:**
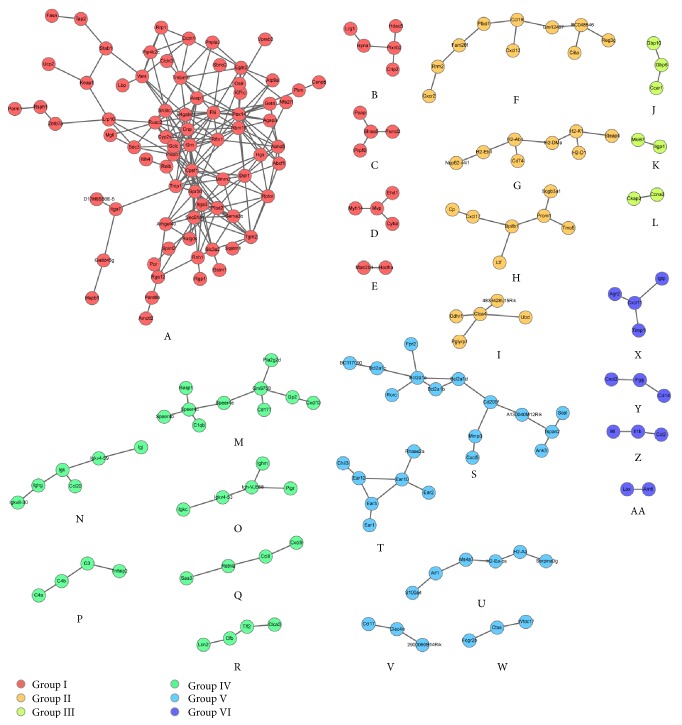
Network analysis. The 205 differentially regulated genes formed 27 clusters (A–AA). According to overlap in function, the clusters were subsequently combined in six groups (I–VI).

**Figure 4:**
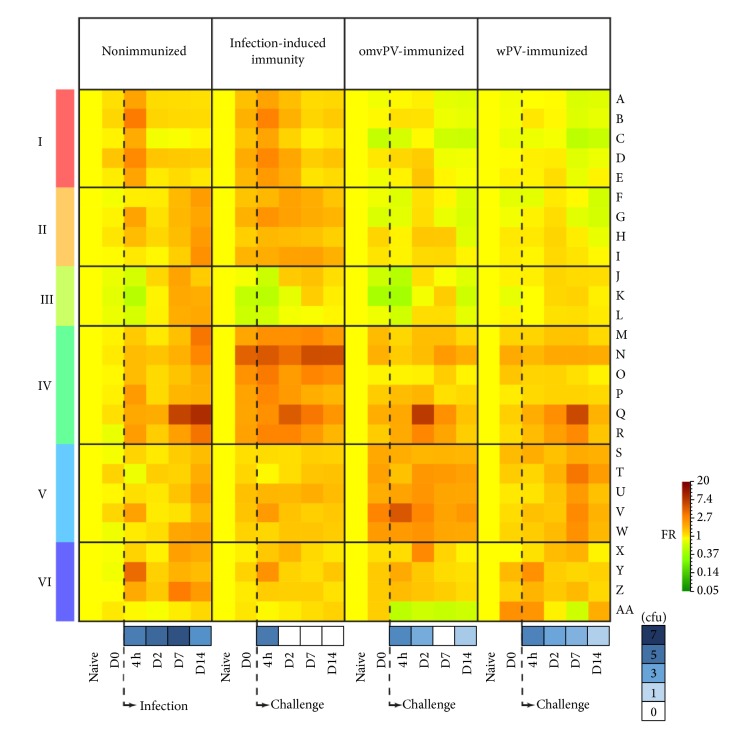
Comparison of pulmonary gene expression profiles following a* B. pertussis* challenge in mice with different pertussis immunity background. Averaged fold changes of 27 clusters (A–AA) of infected nonimmunized mice and challenged mice with infection-induced immunity, omvPV-immunization, or wPV-immunization background are depicted in a time course. Additionally, six groups (I–VI) with similarity in function are shown. The moment at which the* B. pertussis* infection or challenge was performed is depicted as well as the log10 number of colony forming units (cfu) for each time point postchallenge as determined previously [7, 8].

The pulmonary transcriptomes of four individual* B. pertussis* challenge experiments were merged. The immunization and* B. pertussis* challenge scheme of these studies is shown in Figure 1(a). Individual datasets revealed 975 DEGs (FR ≥ 2.0,* p* ≤ 0.001) in one or more datasets (Figure 2). In total, 627, 256, 169, and 280 genes were included in the nonimmunized, omvPV-immunized, and wPV-immunized mice and mice with infection-induced immunity, respectively. Subsequently, we identified DEGs (FR ≥ 2.0,* p* ≤ 0.001) between the datasets for each time point. This second round of identifying DEGs between studies, combined with a “cleanup” by exclusion of functionally less relevant genes, that is, hypothetical or nonprotein-coding genes (Figure 1), left 205 genes for further analysis (Table 1). Coexpression patterns for these 205 genes were determined and visualized in a network analysis (Figure 3). This analysis split the 205 genes into 27 signature clusters (A–AA) ranging in size from 2 to 75 genes (Table 1). The average gene expression was calculated for each gene cluster and visualized in a heatmap (Figure 4).

### 3.2. Function of Clusters and Relation to Pertussis-Vaccinated Background

**Figure 5:**
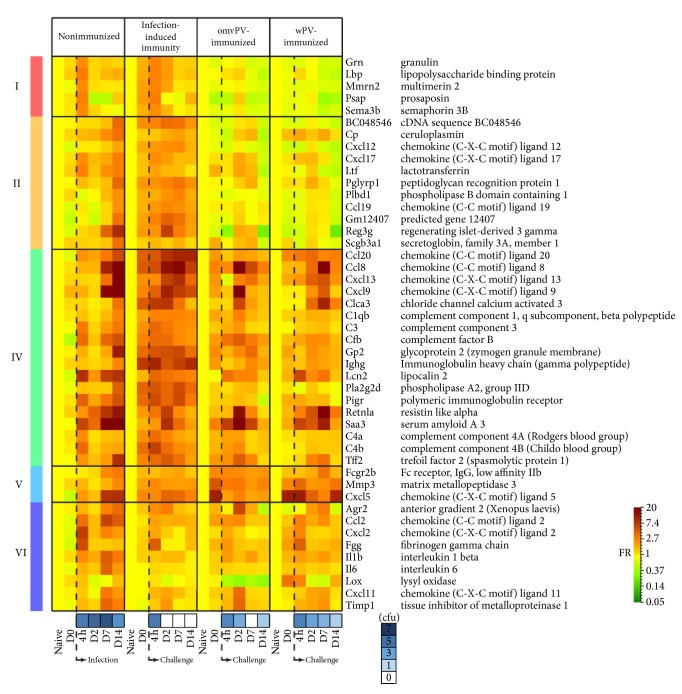
Genes encoding for secreted proteins. Genes encoding for secreted proteins were retrieved from DAVID and illustrated as heatmap for the four pulmonary transcriptome datasets. The genes are clustered according to prevalence in the six function groups (I–VI), which is shown on the left. The moment at which the* B. pertussis* infection or challenge was performed is depicted as well as the log10 number of colony forming units (cfu) for each time point postchallenge as determined previously [7, 8].

To determine the function of the individual clusters, an overrepresentation analysis was performed using DAVID. The different clusters were combined to six groups with overarching biological functions (group I–VI) (Figures 3 and 4). From these molecular signatures, we additionally identified the genes that encode for secreted proteins. Because they may serve as markers that can be analyzed in blood and could be interesting candidates for later application in human studies. In total, 46 genes were identified to code for secreted proteins, which were present in the different groups, except for group III (Figure 5). Gene expression of clusters and single genes were compared with the number of colony forming units (cfu) in the lung (Figures 4 and 5) which were determined previously in primed and unprimed mice [7, 8].

At this point, we are able to isolate different molecular signatures from this analysis based on the gene expression kinetics: first, (i) signatures of local immunity induced by the primary vaccination or infection that are still active or present in the lungs on D0, just before challenge; second, (ii) signatures of infection intensity (the NI mice have the highest number of bacteria in the lungs on 7 days p.i. whereas these are cleared faster in primed mice (Figures 4 and 5); therefore, the kinetics and/or intensity of gene expression of acute phase and proinflammatory proteins that are secreted during colonization could indicate the intensity of infection in the mouse model); finally, (iii) signatures of recall responses of adaptive immunity. These are expressed earlier (within 2 days p.c.) in immunized mice but are absent or expressed later on (7–14 days p.i.) in NI mice. The different, groups (I–VI), clusters (A–AA), and genes encoding secreted proteins will be described hereafter.

#### 3.2.1. Group I: Innate Activation

Clusters A–E were combined to group I and comprised genes involved in general immune responses or related to macrophages and T-cell activation. These clusters were exclusively upregulated in the lungs of NI mice and mice with infection-induced immunity mice 4 hours p.c. Cluster A is the largest cluster, with 75 genes, and included genes involved in cell activation (*Relb, Gadd45g, Lbp, *and* Sbno2*). Lipopolysaccharide binding protein (*Lbp*) is involved in recognition of LPS. Moreover, two integrins (*Itga3, Itga7*) were detected in this group, which are cell membrane proteins but not specific for immune cells.

#### 3.2.2. Group II: Pulmonary Bridging Phase

Clusters F–I are part of group II that was enriched for antigen processing and presentation. These clusters were induced 7–14 days p.c. in NI mice, whereas these clusters were constantly expressed in mice with infection-induced immunity. Cluster G more specifically included genes involved in MHC signaling (*H2-Ab1, H2-D1, H2-DMa, H2-Eb1, *and* H2-K1*). Additionally, group II contains genes coding for proteins that are secreted. These are expressed earlier in mice with infection-induced immunity compared to the mice receiving a vaccination (*Cxcl12, Cxcl17, Ccl19, *and* Pglyrp1*) (Figure 5). Cxcl17 is a mucosal cytokine that attracts lung macrophages [16].

#### 3.2.3. Group III: Cell Cycle and Tissue Remodeling

Group III comprised genes of clusters J–L, associated with the cell cycle, which were only upregulated 7–14 days p.c. in NI mice. This group was marked by high activation of the cell proliferation marker* Mki67*. In addition, this group is comprised of interferon-induced GTPases such as* Gbp6* and* Gbp10*, which are involved in the innate response to protect against a bacterial infection [17] and* Iigp1*. Activation of these genes solely in NI mice suggests enhanced lung cell proliferation as a result of lung tissue damage. Therefore, the absence of this gene expression signature in protected mice might indicate less collateral lung damage as a result of a* B. pertussis* challenge and, accordingly, suggests that recall responses of the adaptive immune system are sufficient.

#### 3.2.4. Group IV: Mucosal and Systemic Adaptive Recall Responses

Clusters M–R were part of group IV of which the genes encode proteins with immunological functions, such as immunoglobulins, complement factors, and acute inflammatory proteins. Clusters N-O both contain genes involved in antibody production. Cluster N is related to IgG production and more active in the lungs of mice with infection-induced immunity compared to omvPV- and wPV-immunized mice. Previously, we showed that pertussis immunization leads to higher total IgG levels compared to infection [18]. The higher expression of these genes in the lungs may suggest that the mice with infection-induced immunity have higher numbers of lung-resident IgG-producing B-cells, especially in combination with the coclustering of these genes with* Ccl20* that attracts CCR6^+^ B-cells. The genes (*Pigr, Igh-VJ558*) in cluster O are specifically related to IgA production [19] and were strongly upregulated in mice with infection-induced immunity from 4 hours to 14 days p.c. This suggests that mice with infection-induced immunity have more intense and faster antibody production in the lungs compared to subcutaneously immunized mice. This was also shown at the functional level by the presence of specific-IgA in the lungs of mice with infection-induced immunity [7] and absence in wPV-mice and omvPV-mice [8]. Local stimulation of the immune system might be critical because the induction of antibody-related genes (*Iga, Pigr*) and mucosal IgA may lead to better protection. Cluster P contains three members of the complement system (*C3, C4a, *and* C4b*). Members of complement systems (*C1qb, Cfb, C3, C4a, *and* C4b*) were expressed most profoundly in the lungs of mice with infection-induced immunity and to a lesser extent in omvPV-immunized mice. Interestingly, these genes were hardly expressed in the lungs of wPV-immunized mice (Figure 5). This suggests that complement-mediated responses, which may contribute to clear* B. pertussis* from the lungs [20], are more prominent in omvPV-immunized mice. Cluster Q showed most pronounced expression in NI mice 7–14 days p.c. but was also present in wPV-mice 7 days p.c. In omvPV-mice and mice with infection-induced immunity, upregulated expression of cluster Q was observed 2 days p.c. This cluster included* Saa3*, part of the acute phase response,* Cxcl9*, that attracts CXCR3^+^ cells, a chemoattractant (*Ccl8*), and resistin-like molecule *α* (*Retnla*) that is important in lung pathology [21]. Notably, the expression of* Saa3* and* Retnla* follows the number of cfu in the lungs (Figure 5). Mice with infection-induced immunity show a limited induction of gene expression for* Saa3* and* Retnla*, which also peaks early, already 2 days p.c. In addition, they show the fastest lung clearance [13]. On the contrary, the wPV-mice and NI mice showed later and more intense gene expression in accordance with prolonged lung clearance. The omvPV-mice revealed earlier expression compared to wPV-mice and NI mice conforming to the lower inflammatory responses observed in omvPV-immunized mice [8]. Both* Saa3* and* Retnla* encode for secreted proteins (Figure 5) and can therefore serve as a predictor of infection intensity when measured in blood.

#### 3.2.5. Group V: Vaccine-Primed Innate Responses

Clusters S–W were part of group V, which included genes associated with myeloid cells. These clusters were mainly upregulated in omvPV-mice 4 hours p.c. and in wPV-mice 7 days p.c. In cluster S, which is moderately expressed in omvPV-mice and wPV-mice, four members were identified belonging to the B cell leukemia/lymphoma 2 related proteins (*Bcl2a1a, Bcl2a1b, Bcl2a1c, *and* Bcl2a1d*), a pathogen recognition receptor (*Fpr2*), a C-type lectin receptor (*Cd209f*), and matrix metallopeptidase 3 (*Mmp3*). Additionally,* Cxcl5*, a neutrophil attractant, was highly expressed before the challenge in the lungs of omvPV-mice and wPV-mice (Figure 5). Notably, this cluster also included the retinoic acid receptor- (RAR-) related orphan receptor C (*Rorc*) that is essential for Th17 cell differentiation [22]. Cluster T included five members (*Ear1, Ear2, Ear3, Ear10, *and* Ear12*) of the eosinophil-associated, ribonuclease A family. Cluster V was intensely upregulated in omvPV-mice 4 hours p.c. It contained* Ccl17* and dendritic cell specific* Clec4n*. Together, these clusters represent an influx or higher proliferation of myeloid cells and DCs that are most profound in the omvPV-mice.

#### 3.2.6. Group VI: Inflammation

Finally, cluster X–AA formed group VI that was enriched for the GO-term inflammatory response. Cluster Y was mainly upregulated 4 hours p.c. This was most intense in nonimmunized mice and the lowest in omvPV-mice. This cluster contained the CD14 marker (*Cd14*) that is involved in LPS recognition, a neutrophil attractant (*Cxcl2*), and fibrinogen (*Fgg*), which is an important attenuator of LPS-mediated responses [23]. Cluster Z included* Ccl2* and the proinflammatory cytokines* Il6* and* Il1b* that were strongly induced in the NI mice and not in the three immunized groups. This indicates that previous exposure to either a vaccine or infection prevents induction of these proinflammatory markers in the lungs upon a* B. pertussis* challenge. Therefore, the presence of these signatures may serve as a marker for mice being unprotected against pertussis (Figure 5). Finally, cluster AA was only present in wPV-immunized mice.

## 4. Conclusions

This meta-analysis revealed molecular signatures specific for immune responses against pertussis. The signatures were measured in the lungs of mice that were previously exposed to either pertussis vaccination or infection. Comparison of the gene expression profiles in the lung of differently treated mice revealed the following:

- Infection, but not subcutaneous vaccination, leads to induction of local immunity in the lungs. This local immunity is characterized by enhanced IgA production and involvement of “trained” pulmonary innate cells [7].
- Responses to pertussis challenge in the lungs of omvPV-mice and wPV-mice were similar in nature, but the omvPV-mice respond slightly faster.
- Gene expressions of complement-mediated responses are more prominent in omvPV-immunized mice than in wPV-mice.
- Genes with unknown function (*Speer4b, Speer4c, *and* Speer4e*) were associated with genes with well-known function (*C1qb, Cd177*) based on their coexpression, providing insight into their potential immunological functions.
- Genes of potentially secreted proteins were identified of which some may serve as markers in blood for analysis of degree of protection (*Cxcl9, Gp2, *and* Pla2g2d*), intensity of infection (*Retnla, Saa3, Il6, *and* Il1b*), or adaptive recall responses (*Ighg, C1qb*). This analysis can be performed by using an ELISA or multiplex immunoassay and, for instance, applied as readout in a human challenge model [12].

## Figures and Tables

**Table 1:** Detailed information on the 27 gene clusters.

Clusters		Number of genes	Included genes
I	A	75	Abcf1, Abhd8, Agap3, Amotl2, Arap1, Arhgef40, Atp9a, B4galnt1, Cenpb, Cldn3, Cnp, Cpsf1, Cyp2s1, D17H6S56E-5, Dctn1, Ddr1, Egln2, Fasn, Flii, Lbp, Gadd45g, Gclc, Get4, Gpr56, Grn, Gstm1, Hgs, Hspb1, Itga3, Itga7, Itih4, Keap1, Kif1c, Lrp10, Mgll, Mmrn2, Nfe2l1, Os9, Pard6b, Parm1, Pex14, Piezo1, Pip4k2c, Pkm, Plbd2, Pnpla2, Por, Ralgds, Rbm19, Relb, Rftn1, Rgp1, Rgs12, Rnh1, Rptor, Rrp1, Rsph1, Rusc2, Sbno2, Sdc3, Sec61a1, Sema3b, Sh3tc1, Slc3a2, Spint2, Sqstm1, Stab1, Tap2, Tgm2, Tmbim6, Tnip1, Ucp2, Vars, Vpreb3, Zbtb7a
	B	5	Crip2, Hdac5, Isyna1, Lrg1, Plxnb2
	C	4	Prpf8, Psap, Psmd3, Shisa5
	D	4	Cyba, Ehd1, Mvp, Myh14
	E	2	Hadha, Man2b1

II	F	10	Ccl19, Ciita, Cxcl12, Cxcr2, Fam26f, Gm12407, Plbd1, Reg3g, Rrm2, BC048546
	G	8	Cd74, H2-Ab1, H2-D1, H2-DMa, H2-Eb1, H2-K1, Nup62-il4i1, Steap4
	H	7	Bpifb1, Cp, Cxcl17, Ltf, Prom1, Scgb3a1, Tmc5
	I	5	Cdhr1, Clca4, Pglyrp1, Ubd, 4833428L15Rik

III	J	3	Ccar1, Gbp10, Gbp6
	K	2	Iigp1, Mki67
	L	2	Ccna2, Ckap2

IV	M	10	Basp1, C1qb, Cd177, Cxcl13, Gm9758, Gp2, Pla2g2d, Speer4b, Speer4c, Speer4e
	N	6	Ccl20, Ighg, Igj, Igk, Igkv4-59, Igkv8-30
	O	5	Igh-VJ558, Ighm, Igkc, Igkv4-53, Pigr
	P	4	C3, C4a, C4b, Tnfaip2
	Q	4	Ccl8, Cxcl9, Retnla, Saa3
	R	4	Cfb, Clca3, Lcn2, Tff2

V	S	14	Ank3, BC117090, Bcl2a1a, Bcl2a1b, Bcl2a1c, Bcl2a1d, Cd209f, Cxcl5, Fpr2, Mmp3, Rorc, Scel, Tspan2, A130040M12Rik
	T	7	Chil3, Ear1, Ear10, Ear12, Ear2, Ear3, Rnase2a
	U	6	Aif1, H2-Aa, H2-Ea-ps, Ms4a7, S100a4, Serpina3g
	V	3	Ccl17, Clec4n, 2900060B14Rik
	W	3	Ctss, Fcgr2b, Wfdc17

VI	X	4	Agr2, Cxcl11, Igtp, Timp1
	Y	3	Cd14, Cxcl2, Fgg
	Z	3	Ccl2, Il1b, Il6
	AA	2	Arntl, Lox
